# Expression of Tryptophan 2,3-Dioxygenase and Production of Kynurenine Pathway Metabolites in Triple Transgenic Mice and Human Alzheimer's Disease Brain

**DOI:** 10.1371/journal.pone.0059749

**Published:** 2013-04-22

**Authors:** Wei Wu, Joseph A. Nicolazzo, Li Wen, Roger Chung, Roger Stankovic, Shisan S. Bao, Chai K. Lim, Bruce J. Brew, Karen M. Cullen, Gilles J. Guillemin

**Affiliations:** 1 Department of Pharmacology, School of Medical Sciences, University of New South Wales, Sydney, New South Wales, Australia; 2 Drug Delivery, Disposition and Dynamics, Monash Institute of Pharmaceutical Sciences, Monash University, Melbourne, Victoria, Australia; 3 Cornea and NSW Eye Bank, The University of Sydney, Sydney, New South Wales, Australia; 4 Menzies Research Institute Tasmania, The University of Tasmania, Hobart, Tasmania, Australia; 5 Discipline of Pathology, The University of Sydney, Sydney, New South Wales, Australia; 6 MND and Neurodegenerative Disease Research Group, Macquarie University, North Ryde, New South Wales, Australia; 7 St Vincent's Centre for Applied Medical Research, Darlinghurst, New South Wales, Australia; 8 Departments of Neurology and HIV Medicine, St. Vincent's Hospital, Darlinghurst, New South Wales, Australia; 9 Discipline of Anatomy and Histology, The University of Sydney, Sydney, New South Wales, Australia; The Mental Health Research Institute, University of Melbourne, Australia

## Abstract

To assess the role of the kynurenine pathway in the pathology of Alzheimer's disease (AD), the expression and localization of key components of the kynurenine pathway including the key regulatory enzyme tryptophan 2,3 dioxygenase (TDO), and the metabolites tryptophan, kynurenine, kynurenic acid, quinolinic acid and picolinic acid were assessed in different brain regions of triple transgenic AD mice. The expression and cell distribution of TDO and quinolinic acid, and their co-localization with neurofibrillary tangles and senile β amyloid deposition were also determined in hippocampal sections from human AD brains. The expression of TDO mRNA was significantly increased in the cerebellum of AD mouse brain. Immunohistochemistry demonstrated that the density of TDO immuno-positive cells was significantly higher in the AD mice. The production of the excitotoxin quinolinic acid strongly increased in the hippocampus in a progressive and age-dependent manner in AD mice. Significantly higher TDO and indoleamine 2,3 dioxygenase 1 immunoreactivity was observed in the hippocampus of AD patients. Furthermore, TDO co-localizes with quinolinic acid, neurofibrillary tangles-tau and amyloid deposits in the hippocampus of AD. These results show that the kynurenine pathway is over-activated in AD mice. This is the first report demonstrating that TDO is highly expressed in the brains of AD mice and in AD patients, suggesting that TDO-mediated activation of the kynurenine pathway could be involved in neurofibrillary tangles formation and associated with senile plaque. Our study adds to the evidence that the kynurenine pathway may play important roles in the neurodegenerative processes of AD.

## Introduction

**Figure 1:**
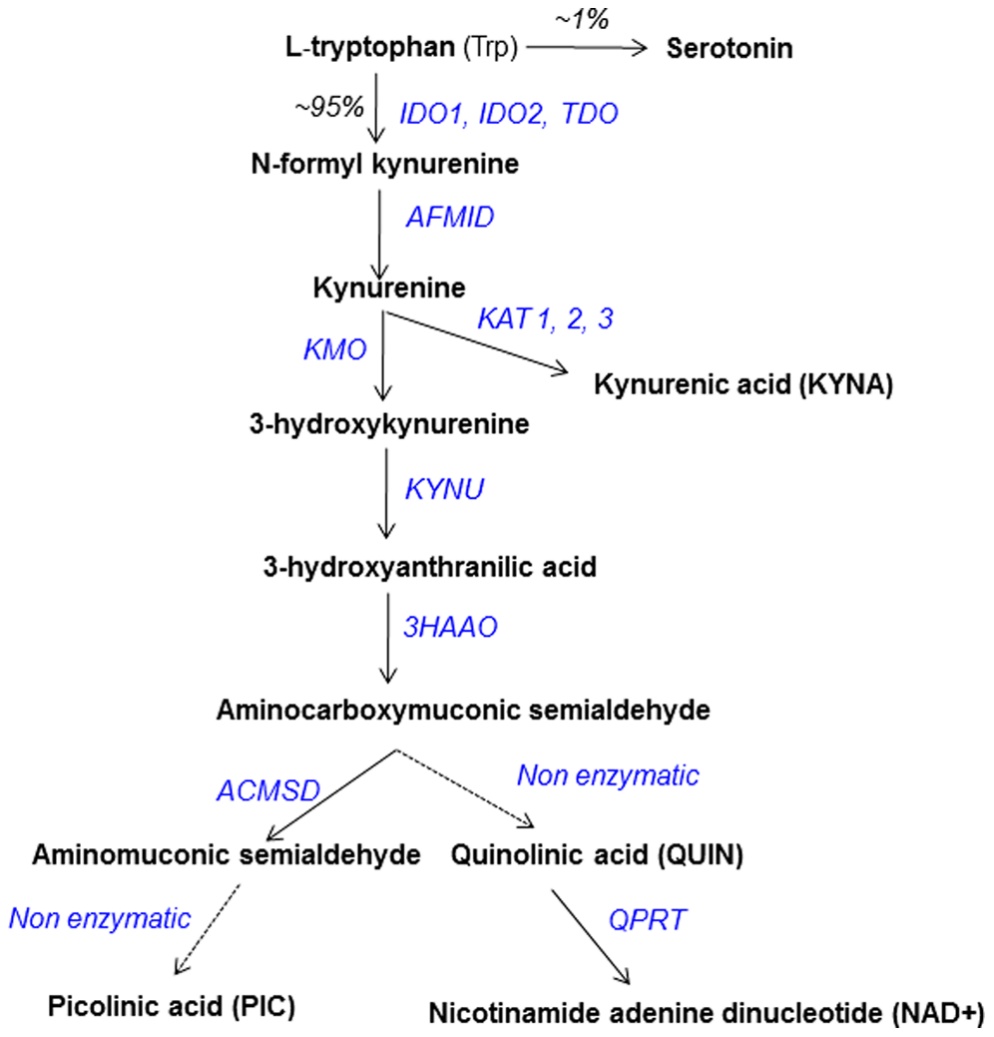
Simplified schematic of the kynurenine pathway (KP). IDO-1, indoleamine dioxygenase; TDO, tryptophan dioxygenase; AFMID, arylformamidase; KAT 1–3, kynurenine amino transferase 1, 2 and 3; KMO, kynurenine 3-hydroxylase; KYNU, kynureninase; 3HAAO, 3-hydroxyanthranilic acid oxidase; ACMSD, amino-carboxymuconate-semialdehyde decarboxylase; QPRT, quinolinate phosphoribosyltransferase.

During neuroinflammation, 95% of the cerebral pool of the essential amino acid tryptophan (Trp) is catabolized through the kynurenine pathway (KP) leading to the formation of several neuroactive metabolites (**Fig. 1**). The downstream metabolites of the KP and especially quinolinic acid (QUIN) an agonist of NMDA receptors [1], [2], have been implicated in the pathophysiology of several brain diseases.

The initial step of the KP is mainly regulated by two key enzymes: indoleamine 2,3-dioxygenase 1 (IDO-1) and tryptophan 2,3-dioxygenase (TDO), which differ in their tissue localization and regulation [3]. IDO-1 is widely expressed in all tissues and is involved in the metabolism of Trp [4], [5]. IDO-1 is activated by pro-inflammatory cytokines and other molecules [6]. IDO-1 plays a key role in the regulation of the CNS immune response [7]. TDO is predominantly expressed in the liver but is also present in the brain [8]. TDO is responsible for systemic metabolism of Trp and is activated by cortisol [9] and L-Trp [10]. In mouse, TDO expression is involved in the development of the brain and nervous system [11].

AD is an age-related neurodegenerative disorder characterised by neuronal loss and dementia. The pathological mechanisms underlying this disease are still controversial, however, there is growing evidence implicating KP metabolites in the development and progression of AD. Some observations include an increased ratio of 3-hydroxykynurenine to Trp in the serum of AD patients [12] and accumulation of QUIN in the brain of AD patients [13]. We have demonstrated that IDO-1 expression and QUIN production are increased in AD hippocampus [14] and that QUIN leads to tau hyperphosphorylation in human cortical neurons [15]. Another recent study showed that IDO-1 is up-regulated in the brains of AD and is associated with neurofibrillary tangles (NFT) and β-amyloid (Aβ) plaques [16]. All together, these studies strongly suggest that the KP is involved in the neurodegenerative processes of AD.

The purpose of this study was to characterize the KP metabolic profile and assess TDO and IDO-1 expression in the brains of AD patients and in an AD mouse model. We use triple-transgenic AD (3xTg AD) mice, a relevant model exhibiting both Aβ and tau pathologies [17]. This model provides a unique opportunity for demonstrating the importance of the KP and TDO during the progression of AD.

We studied three age groups of 3xTg AD and wild type mice. We investigated the expression of TDO and IDO-1 in different brain regions using real-time RT PCR, immunohistochemistry and Western blotting and TDO/IDO-1 activity using HPLC and gas chromatography-mass spectrometry (GC-MS). The expression of TDO and IDO-1 protein was then assessed in the hippocampus of AD patients and controls. Finally, the co-localization of TDO with QUIN, NFTs and Aβ deposits was assessed to discern the roles of TDO-initiated KP in the pathological progression of AD.

## Materials and Methods

### Ethic

Both Human and animal studies, have the appropriate Ethic approvals.

#### Animal

Sections and tissue were obtained from the brains of 3xTg and WT mice under procedures approved by the Monash Institute of Pharmaceutical Sciences Animal Ethics Committee and which were performed in accordance with the Australian National Health and Medical Research Council guidelines for the care and use of animals for scientific purposes.

#### Human

Post mortem brain tissue from 4 AD cases aged 63 to 86 years (75±11 years) and 4 age and sex-matched controls were obtained from the Discipline of Pathology, University of Sydney, Australia. Written informed consents have been obtained. The study was approved by the Human Ethics Committee of the University of New South Wales."

### 3xTg AD mice

The 3xTg mouse, which possesses the transgenes PS1M146V, APPSwe, and tauP301L transgenes, was originally provided by Prof Frank LaFerla (University of California, Irvine) [17]. Brains from 2–4 month, 6–8 month and 10–12 month-old 3xTg AD mice (3 to 6 mice per group) and age matched 129C57BL/6 WT mice were removed following perfusion with ice-cold PBS. Hemispheres were embedded in OCT medium (TissueTek, CA, USA) then fresh frozen for immunohistochemistry. For real time RT-PCR and biochemistry assays, the other hemisphere was dissected into hippocampus, cerebellum, cortex and remaining brain regions (including basal ganglia and brainstem), and immediately frozen in liquid nitrogen. In addition, the hippocampus and cerebellum from another two groups of 8-month 3xTg AD or WT control mice (n = 3 each) were placed in 600 µl RIPA lysate buffer with protease inhibitor (1 tablet per 50 ml), and snap frozen for Western blotting.

### Real-time RT PCR

RNA was extracted from mouse brain tissue using Purelink RNA mini kit (Invitrogen, CA, USA). cDNA was synthesized from up to 1 µg of total RNA of each brain sample using superscript III VILO cDNAsyn kit (Invitrogen). A 5 µl sample of cDNA product (equivalent to 5 ng RNA) was used per 20 µl reaction mix in each well, with 10 µl express SYBER green qPCR super mix universal (Invitrogen), and 0.6 µl of each primer (10 µM) to bring to 300 µM final concentration per well. Each sample was duplicated and the PCR controls were included in each run, in which a 5 µl aliquot of West Nile virus-infected WT mouse brain (+) and water (−) was added instead of the sample. The oligonucleotide sequences of the primers used for RT-PCR are listed in Table 1.

**Table 1:** Primer sets for murine KP enzymes.

Genes	Forward	Reverse
HPRT	GGACCTCTCGAAGTGTTGGA	TTGCGCTCATCTTAGGCTTT
RPL13	GAGGTCGGGTGGAAGTACCA	TGCATCTTGGCCTTTTCCTT
TDO	TGCTCAAGGTGATAGCTCGGA	AGGAGCTTGAAGATGACCACCA
IDO-1	TGTGAATGGTCTGGTCTC	CTGTGCCCTGATAGAAGT
3HAAO	TTCAGCCTCATTGCATCT	GACAGTGTAGGGCTATGG
ACMSD	GAATAAATGCTGACCCAACA	TTCATCCATCCTTCCAGAC
QPRT	CCGGGCCTCAATTTTGCATC	GGTGTTAAGAGCCACCCGTT

An Mx3500P Real-Time PCR instrument (Stratagene, NSW, Australia) was used to perform real time RT-PCR. After incubations for 2 min at both 50°C and 95°C, DNA amplification occurred over 40 cycles (about 10 min) at 15 sec incubation at 95°C, followed by a 60-s incubation at 60°C. The melting curve was run at the end to confirm the identity and purity of the PCR. mRNA levels were expressed relative to the mean of 2–4 month WT hippocampus samples using the delta C_T_ method with varying efficiencies, after adjustment according to levels of the reference housekeeping genes, HPRT and RPL13.

### Western blotting

Using a Precelly 24-dual homogenizer (PEQLAB, Erlangen, Germany), hippocampal and cerebellar tissues were homogenized in RIPA lysate buffer (Sigma, MO, USA) with proteinase inhibitor (Roche, NSW, Australia) at 1 tablet per 50 ml. After centrifugation at 12,000 rpm for 10 min (at 4°C), supernatants were collected. Protein concentrations were determined using a Pierce BCA protein assay kit (Thermo Scientific, VIC, Australia). A 50 µg sample (for IDO1 detection) or 20 µg sample (for TDO detection) of denatured lysate from each sample was loaded into 10% NuPAGE Bis-Tris gel (Invitrogen, CA, USA) along with molecular mass markers-PageRuler TM (Thermo Scientific, VIC, Australia), and fractionated by electrophoresis. The protein was transferred onto a polyvinylidenedifloride membrane (Invitrogen, CA, USA). The membrane was blocked with 5% BSA in Tris-buffered saline plus 0.05% Tween20 (TBS-T) for 1 hour at room temperature before probing the membrane with a 1∶1500 dilution of rat anti-mouse IDO1 mAb (Biolegend, CA, USA) or a 1∶1000 dilution of rabbit anti-TDO2A pAb (a gift from Dr Christine Miller, Johns Hopkins University, USA) overnight at 4°C. After three washes with TBS-T, the membrane was incubated with HRP-conjugated anti-rat IgG (GE healthcare, UK) or HRP-conjugated anti-rabbit IgG (Millipore, MA, USA), respectively, for 2 h at room temperature. After another three washes, the membrane was incubated with ECL (Roche, NSW, Australia) for 6 min, the signal was visualized using Gbox (Syngene, Cambridge, UK) and the Gensnap program. For a loading control, antigen-antibody complex was stripped off with Stripping buffer (Thermo Fisher Scientific, VIC, Australia), and re-probed with anti-mouse GAPDH mAb (1∶10000, Sigma, MI, USA) overnight at 4°C, and followed by HRP-conjugated anti-mouse IgG (Upstate, USA). Band density was quantified using GeneTools analysis software (Syngene, Cambridge, UK) and normalized to the loading control.

### Analytical biochemistry

Snap frozen sections of hippocampus, cerebellum, cortex and remaining regions of each brain from both 3xTg AD and WT mice were weighed and homogenised using Precelly 24-dual homogenizer (in accordance with manufacturer instruction) with addition of 0.1 M perchloric acid in order to deproteinized the tissue. Samples were centrifuged at 12,000 rpm for 5 min at 4°C, and the supernatants filtered through a 0.45 µm ACRODISC® CR 4 mm PTFE syringe filter (Waters Cooperation, MA, USA). Samples were concentrated using a Savant SpeedVac® Concentrator (Thermo Scientific, MA, USA) and resuspend in 100 µl ultrapure water prior to HPLC analysis or gas chromatography-mass spectrometry (GC-MS) to detect Trp, KYN, KYNA, QUIN and PIC, as described previously [18].

### Immunohistochemistry

#### Mouse

Fresh frozen OCT medium embedded 3xTg and WT mouse brains were cut into 7 µm-thick sections and mounted on Superfrost Plus slides (Allegiance, IL, USA). For TDO or IDO-1 staining, sections were fixed in acetone for 10 min and, after air-drying, the sections were incubated in TNT (0.1 M Tris pH 7.5, 0.15 M NaCl, 0.01% w/v Tween 20) for 10 min before blocking endogenous peroxidase activity in 3% w/v H_2_O_2_ for 5 min. Endogenous biotin and avidin activity was blocked by using Biotin Blocking kit (DAKO, Glostrup, Denmark), followed by a 15 min blocking of non-specific binding with TNT containing 0.3% (w/v) blocking agent (TNB) (Perkin Elmer, MA, USA). The solution was removed and sections were incubated with rabbit anti-TDO2-B pAb (1∶250, a gift from Dr. Christine Miller, Johns Hopkins University, USA) or rat anti-mouse IDO1 mAb (1∶200) for 2 hours at room temperature. After three washes with TNT, sections were incubated in DAKO EnVision Polymer/HRP G/2 double System (DAKO, Glostrup, Denmark) for 30 min, followed 3,3′diaminobenzidine (liquid DAB^+^, DAKO, Glostrup, Denmark) for 5 min. The tissue was counterstained with hematoxylin, dehydrated through graded alcohol solutions and xylene, and mounted in Fastmount (Lomb Scientific, Sydney, Australia).

For immunohistochemical identification of neurons, microglia/macrophages and astrocytes, the following primary antibodies were used: monoclonal NeuN for neurons (Millipore, MA, USA), monoclonal GFAP for astrocytes (Invitrogen), and FITC conjugated BS-Isolectin-B_4_ for microglia (Sigma, MI, USA). Double labelling of TDO2-B and NeuN, GFAP or BS-Isolectin-B_4_ was carried out using the following procedure. Sections were fixed in 4% paraformaldehyde for 15 min at 4°C and then washed in PBS three times, after which they were incubated in glycine (100 mM) for 5 min to quench the fixation reaction. The sections were then permeabilized in 0.5% of triton X-100 for 10 min at room temperature, followed by non-specific blocking as above. Sections were then incubated with TDO2-B (1∶100), NeuN (1∶100), or GFAP (1∶100) at 4°C overnight. After rinsing in TNT 3 times, the sections were incubated for 1 h at room temperature with the following secondary antibodies: donkey anti-rabbit IgG Alexa-594 (1∶1000, Invitrogen), donkey anti-mouse IgG Alexa-488 (1∶1000, Invitrogen) or FITC-BS-Isolectin-B_4_ (1∶50). Following three rinses in TNT, sections were counterstained with DAPI for 5 min at room temperature. Following another three washes, the slides were mounted with Aqua Poly Mount (Polysciences, US) and visualized by confocal microscopy (Olympus FV1000, Germany).

#### Human

Post mortem brain tissue from 4 AD cases aged 63 to 86 years (75±11 years) and 4 age and sex-matched controls were obtained from the Discipline of Pathology, University of Sydney, Australia. Formalin fixed blocks from the hippocampal region of AD and age-matched controls were embedded in paraffin and cut at 5 μm sections on superfrost plus slides as before. For TDO/IDO-1 staining, sections were deparaffinised in xylene for 20 min and rehydrated through graded alcohol treatment. Antigen retrieval was performed through the use of Target Retrieval solution pH 6 (DAKO, Glostrup, Denmark) and autoclaving at 121°C for 20 min. Endogenous peroxidase was blocked using 3% w/v hydrogen peroxide for 5 min and endogenous biotin was blocked as described previously. After incubation with 10% horse serum for 30 min to block non-specific binding, sections were incubated with rabbit anti-TDO2-B (1∶250) or mouse anti-IDO-1 primary antibodies (1∶200, gift from Prof Osamu Takikawa, National Center for Geriatrics and Gerontology, Japan) for 2 hours at room temperature. After 3 rinses, sections were incubated with relevant secondary antibodies–biotinylated goat-anti rabbit or mouse (Vector Laboratories, Burlingame, USA 1∶200) for 30 min at room temperature followed by a 30 min treatment with avidin-biotin-complex (ABC) elite (Vector). Labelling was visualized with liquid DAB and sections were counterstained and mounted as above. Double fluorescent labelling for TDO2-B and NeuN, GFAP or HLA-DR (1∶40, Dako, Glostrup) was performed on antigen-retrieved sections for identification of neurons, astrocytes, and microglia/macrophages, respectively.

Duel-labelling for TDO with QUIN, and TDO with NFT-tau or Aβ deposits was performed using rabbit anti-TDO2-B pAb (1∶100) and mouse anti-QUIN mAb (1∶100, Millipore, MA, USA), mouse anti-AT8 mAb (phosphorylated tau, 1∶100, Pierce Biotechnology, Rockford, USA) or mouse anti-4G8 mAb (amino acid residues 17–24 of Aβ protein, 1∶200, Millipore, MA, USA). The double staining was carried out as above, with the exception that sections used for detecting QUIN were given a further tyramide signal amplification (TSA, Perkin Elmer, MA, USA). For 4G8 staining slides, the antigen retrieval consisted of an additional 15 min treatment in 99% w/v formic acid. Negative controls for non-specific staining included replacement of the primary antibody by normal rabbit or mouse IgG.

### Quantitative analysis

The mouse and human brain sections were examined with a Leica Q500 microscope. TDO stained sections in mouse brain were quantified by measuring the mean intensity of all the DAB stained areas of each micrograph using Image-Pro Plus 5.1 software (Diagnostic Instruments, USA), and the data were expressed as mean density, as determined by computer-aided planimetry. TDO/IDO-1 staining in human AD brain were counted individually and converted to the number of TDO^+^/IDO-1^+^ cells per mm^2^. A minimum of 10 randomly selected fields per section from each case was analysed. For measurement and counting, a 20x objective was used.

#### Statistics

Statistical analysis was performed using a two-way analysis of variance (ANOVA) test with a Bonferroni post-hoc test or Student's t test (Windows Prism5 program). Comparisons with p values <0.05 were considered as statistically significant. All data are presented as mean ± SEM, unless stated otherwise.

## Results

### TDO and IDO-1 mRNA expression in 3xTg AD mice

**Figure 2:**
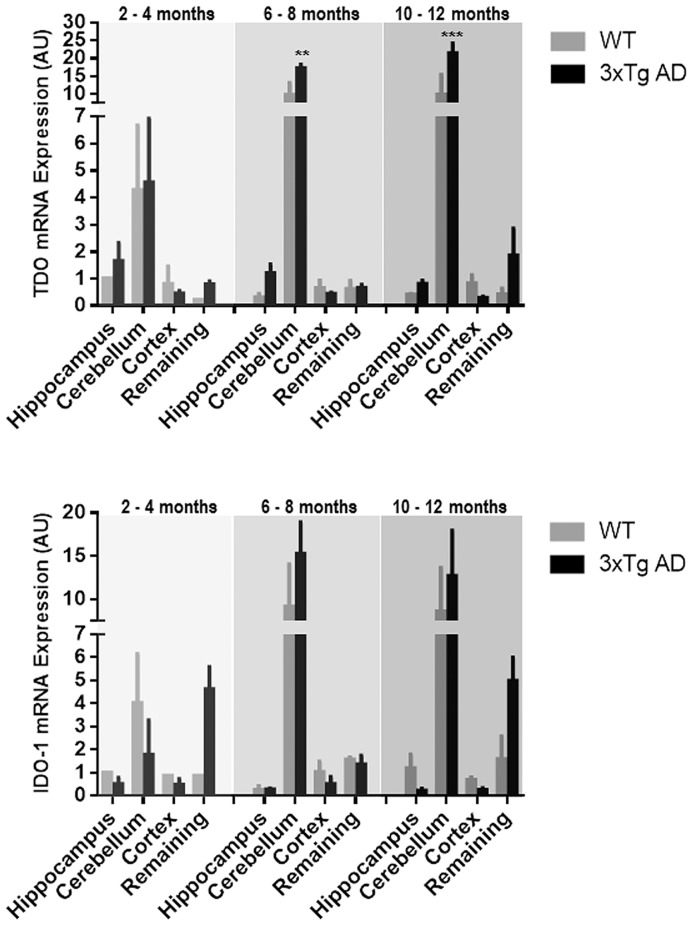
Expression of TDO and IDO-1 mRNA in the hippocampus, cerebellum, cortex and remaining brain regions in 3xTg AD and WT (grey bars) and 3xTg AD (black bars) mice at of 2–4 months, 6–8 months and 10–12 months. Data are presented as mean ± SEM (n = 3–6). **p<0.01, ***p<0.001, in comparison to its counterpart, WT.

The mRNA expression of TDO and IDO-1 in various brain regions of different age groups of 3xTg AD mice was compared to age matched WT controls (**Fig. 2**). A minor increase in the mRNA of IDO1 levels, but not for TDO mRNA, was found in the hippocampus of 3xTg AD mice (2–4, 6–8 and 10–12 month). Elevated levels of TDO and IDO-1 mRNA was observed in 6–8 month 3xTg AD cerebellum compared to age matched controls (1.8-fold and 1.7-fold, respectively) and in 10–12 month mice (2.2-fold and 1.5-fold, respectively), but the difference was statistically significant only for TDO mRNA expression (p<0.01 for 6–8 month, p<0.001 for 10–12 month).

### TDO and IDO-1 protein detection in 3xTg AD mice vs. age matched WT controls

**Figure 3:**
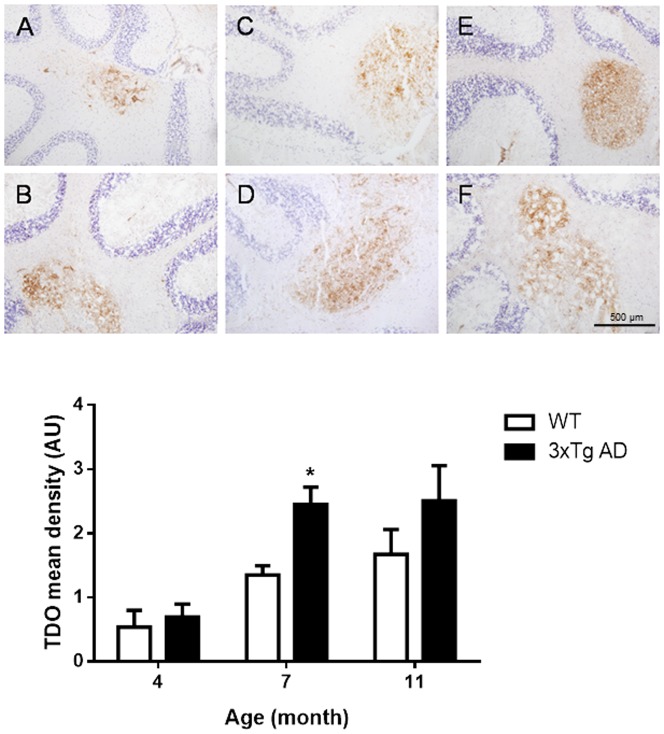
Immunohistochemical detection of TDO in the white matter of cerebellum in 4-month (A), 7-month (C) and 11-month (E) WT mice, compared to the same regions in 4-month (B), 7-month (D) and 11 month (F) 3xTg AD mice. (G) Corresponding graphs of image analysis of density of TDO-immunoreactivity in 4, 7 and 11-months WT (white bars) and 3xTg AD (black bars) mice brains; mean ± SEM (n = 3) *p<0.05, in comparison to its counterpart, WT.

We used immunohistochemistry to confirm elevated protein levels of TDO in the brains of 3xTg mice. Most TDO positive cells were detected in deep nuclei of the cerebellum, pons, hippocampus and the midbrain in WT and 3xTg AD mice (**Fig. 3**). The level of TDO expression increased with age in both mouse strains, however higher TDO immunoreactivity was observed in 6–8 month groups of 3xTg AD mice compared to age matched WT controls (1.8-fold for 7 month, 1.5-fold for 11 month), with significant difference showing in 7-month 3xTg AD mice compared to age matched WT controls (p<0.05) (**Fig. 3**). Similar levels of IDO-1 immunoreactivity were also observed in the endothelial cells in choroid plexus in all age groups of WT and AD mice (data not shown). This may be caused by the low sensitivity of IDO-1 mAb we used for IDO-1 immunohistochemistry labelling in this study.

**Figure 4:**
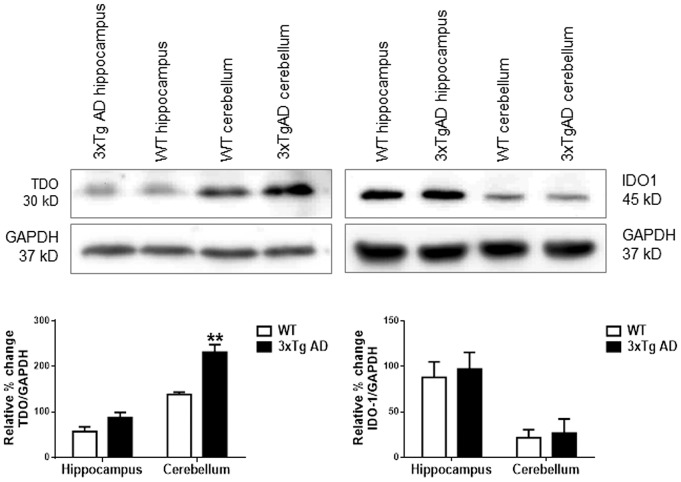
The upper panel shows the representative bands of Western blots for TDO (30 kDa) and IDO1 (45 kDa) proteins in the hippocampus and cerebellum of 8-month WT and 3xTg AD mice. GAPDH was used as a loading control. The lower panel shows the quantitative comparison expressed as relative percentage change of TDO/GAPDH or IDO1/GAPDH ratio in both regions of 8-month WT (white bars) and 3xTg AD (black bars) mouse brains. Data are presented as mean ± SEM (n = 3), **p<0.01, in comparison to its counterpart, WT.

**Figure 5:**
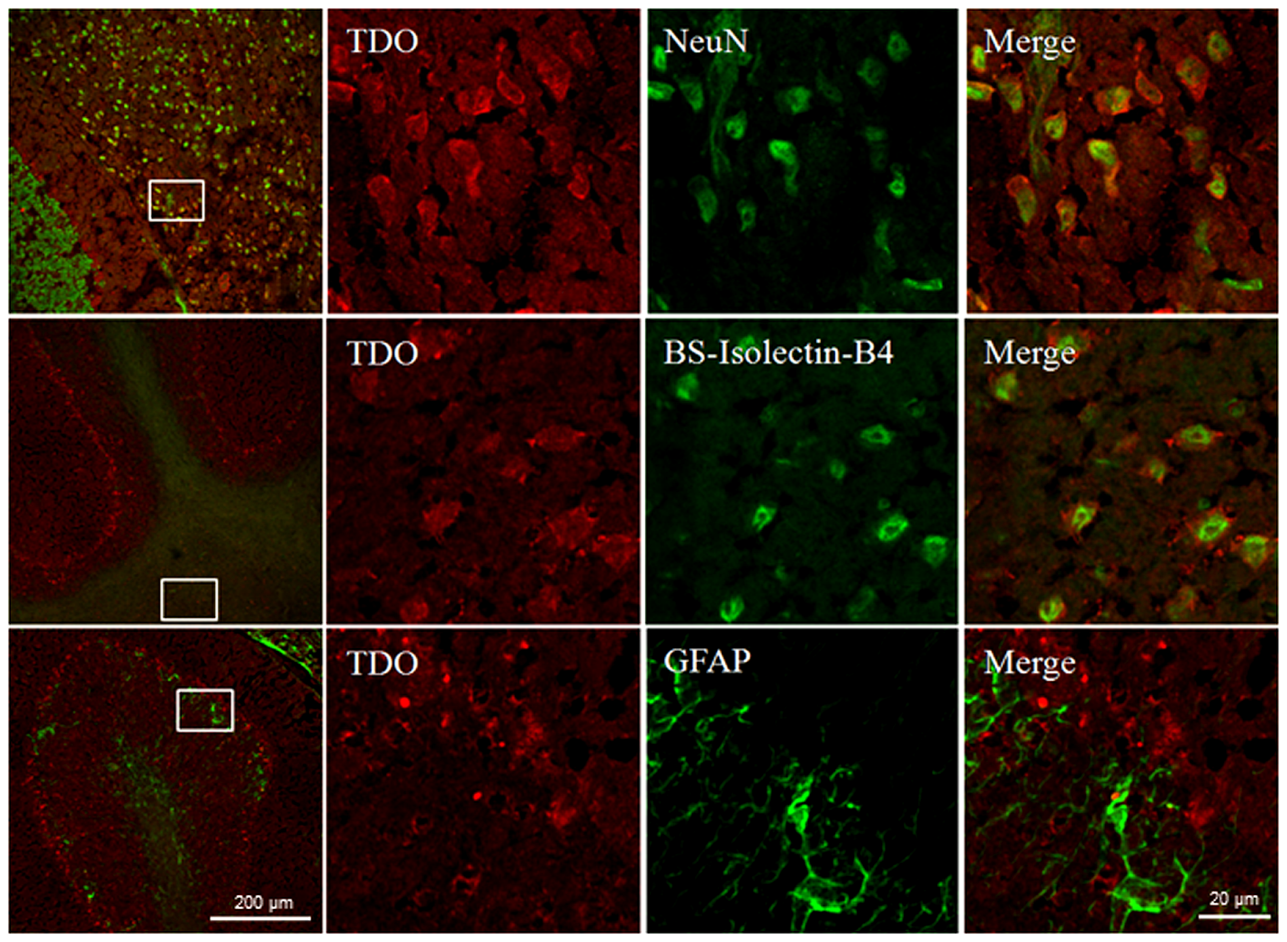
Immunohistochemical localization of TDO in the cerebellum of 3xTg AD mice. TDO was located mainly in neurons (NeuN+) and microglia (BS-Isolectin-B4+), with minor expression in astrocytes (GFAP). Columns 2, 3 and 4 show the higher magnification views of the boxed areas in the first column (merged image).

The highly expressed TDO protein in adult AD mice in particular, in the cerebellum region was further confirmed by western blot analysis. Figure 4 demonstrates significantly higher levels of TDO but not IDO1 protein were detected in the cerebellum of 8-month-old 3xTg AD, compared to those in same age WT mice (p<0.01). Moderate but not significant increased levels of TDO and IDO1 proteins were also appeared in the hippocampus of same age of 3xTg AD brains (**Fig. 4**, lower panel). TDO stained almost all neurons (NeuN) and microglia (BS-Isolectin-B4), but its staining was observed in only some astrocytes (GFAP). The staining of TDO in neurons, microglia and astrocytes was cytoplasmic (**Fig. 5**).

### Quantification of Trp metabolites: KYN, KYNA, QUIN and PIC

**Figure 6:**
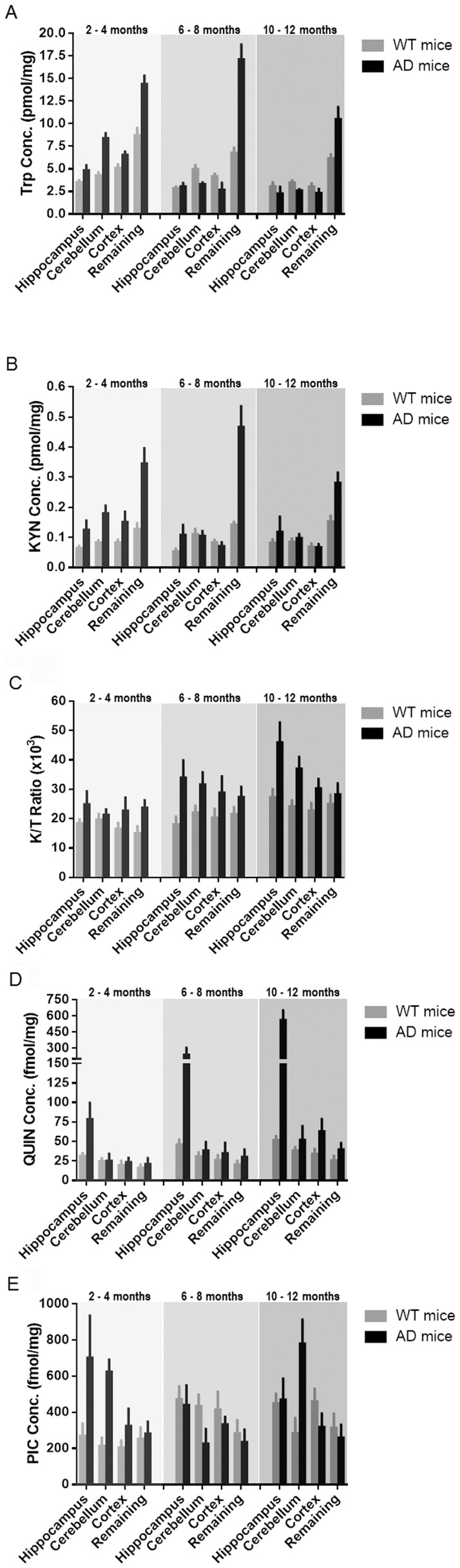
(A) TRP and (B) KYN concentrations quantified using HPLC; (C) K/T ratio depicting the expression of KP activation; (D) QUIN and (E) PIC production quantified using GC/MS in the hippocampus, cerebellum, cortex and remaining brain regions of 2–4 month, 6–8 months and 10–12 months 3xTg AD mice (black bars), compared to WT control mice (grey bars) values in the same regions. Bars represent mean ± SEM, n = 4.

To determine the activity of IDO-1/TDO, we measured the levels of Trp and its metabolite KYN by HPLC (**Fig. 6A**) and quantified the downstream metabolites QUIN and PIC by GC/MS (**Fig. 6B**). We found increased Trp degradation (reflected by K/T ratio) in the hippocampus, cerebellum and cortex in the 3xTg AD compared to WT mice. This increase was also associated with increase in age of the AD mice (**Fig. 6C**). No KYNA was detected in any of the assessed brain regions in 3xTg AD or WT mice (Data not shown) likely due to the detection limit of the HPLC.

Very interestingly, QUIN was found predominantly in the hippocampus in 3xTg AD mice and increased with age (**Fig. 6D**). In addition, we also found an increasing trend of QUIN production with age in both AD and WT mice where this increase was more profound in the AD mice group. However, this observation did not reach statistical significant (p<0.043). High levels of PIC were detected in the hippocampus and cerebellum in young adult (2–4 months) 3xTg AD mice. However, a decreasing trend of neuroprotective, PIC concentration level in the hippocampus and cerebellum of older adult AD mice was observed when compared to WT counterpart (**Fig. 6E**).

### mRNA expression of downstream metabolic enzymes of the KP

**Figure 7:**
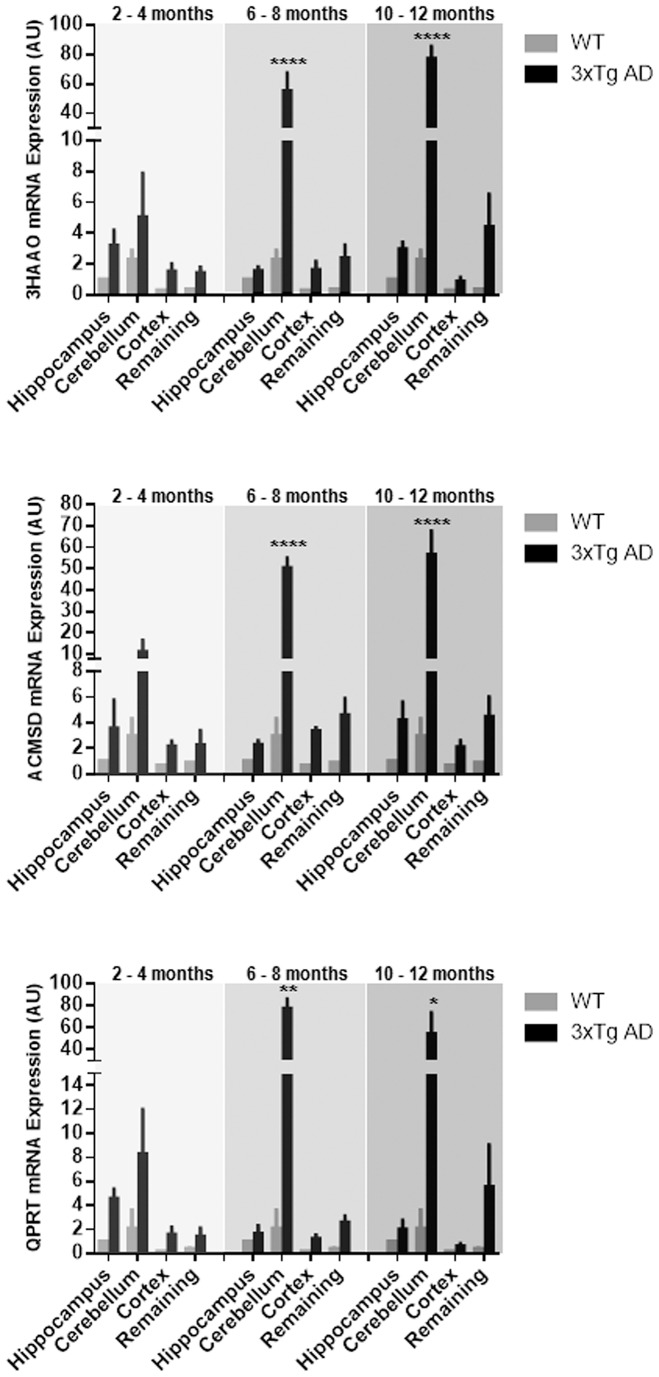
3-HAAO, ACMSD and QPRT mRNA expression in the hippocampus, cerebellum, cortex and remaining regions of 2–4 months, 6–8 months and 10–12 months 3xTg AD mice (black bars), compared to 2–4 months WT control mouse (grey bars) values in the same regions. Bars represent mean ± SEM, n = 3–6, *p<0.05, **p<0.01, ****p<0.0001, in comparison to its counterpart, WT.

We also measured the mRNA expression of three downstream metabolic enzymes of KP involved in QUIN and PIC production and/or catabolism: 3-hydroxyanthranilic acid oxidase (3HAAO), α-amino-α-carboxymuconate-semialdehyde decarboxylase (ACMSD), and quinolinate phosphoribosyltransferase (QPRT) in 3xTg AD mouse brains at various ages (**Fig. 7**). Quantitative real-time PCR revealed that 3HAAO, ACMSD and QPRT enzymes were expressed in various regions of 3xTg AD mice at all age groups tested, with markedly higher expression in the cerebellum, followed by the hippocampus and remaining brain regions. Compared to WT controls, significant differences (p<0.0001) were detected in the cerebellum of 6–8 months and 10–12 months AD brain for mRNA expression of 3HAAO and ACMSD, significantly increased mRNA expression of QPRT was also shown in the cerebellum of 6–8 months (p<0.01) and 10–12 months (p<0.05) for AD mice.

### Semi-quantification of TDO and IDO-1 expression in human AD hippocampus

**Figure 8:**
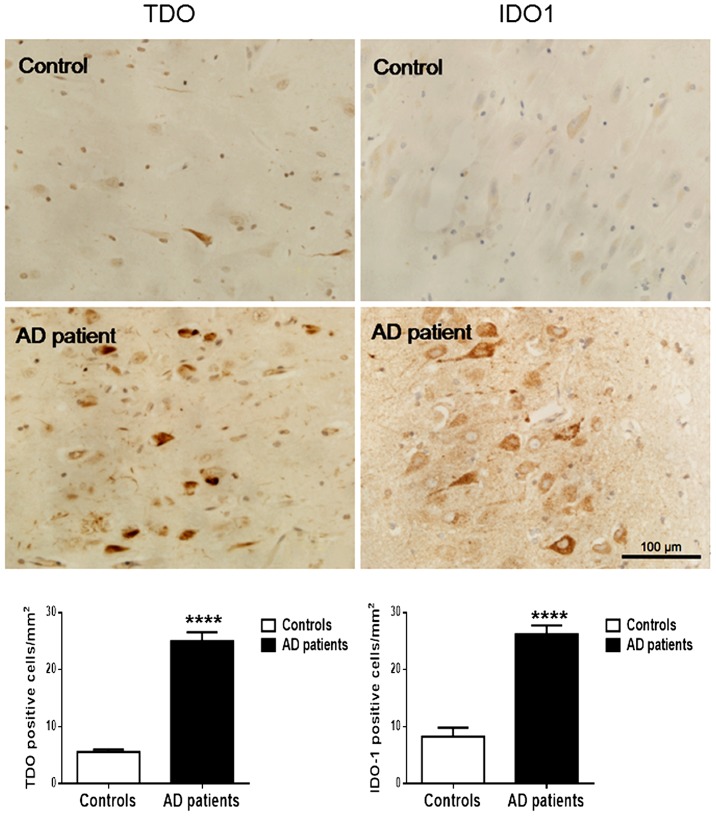
Immunohistochemical detection of TDO/IDO-1 in the CA1 and CA3 regions of hippocampus in AD patients and age and sex-matched controls. Corresponding graphs of image analysis of TDO/IDO-1 immunoreactivity in human controls (white bars) and AD patients (black bars). Columns and bars represent mean ± SEM, n = 4, ****p<0.0001, in comparison to its counterpart, WT.

To further investigate the high expression of TDO found in the AD mouse brain, we performed an immunohistochemical study for TDO and IDO-1 in the hippocampus of four AD patients with age and sex matched controls. Most of the staining for TDO and IDO-1 appeared in the hippocampus proper and the subiculum. Significantly higher TDO and IDO-1 immunoreactivity was observed in AD patients compared to age and sex matched controls (4.5-fold, p<0.0001; 2.9-fold, p<0.0001 respectively) (**Fig. 8**). Moderate IDO-1 staining was also observed in the same regions in one of the age-matched controls (age 86 years). Intensive IDO-1 staining was also observed around the blood vessels in the stratum radiatum of AD brain (data not shown). TDO was strongly expressed in the cytoplasm of neurons (NeuN) and astrocytes (GFAP), with no HLA-DR- positive (microglia/ macrophages) cells, albeit microglia/macrophage were TDO immunopositive in the mouse model (**Fig. 5**).

### Co-localization of TDO with QUIN, tau and Aβ in human hippocampus

**Figure 9:**
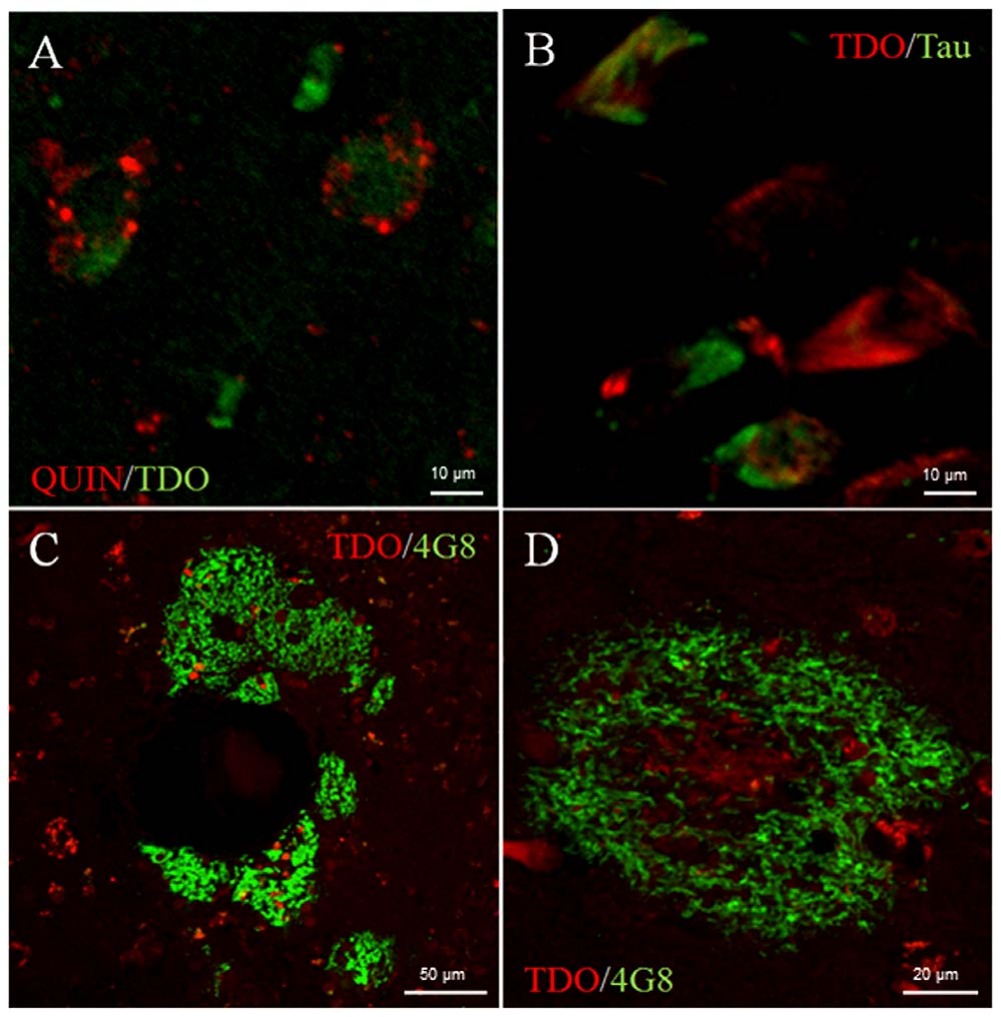
Double-labelling for QUIN and TDO (A), TDO with tau (B) and TDO with Aβ deposit (4G8 Ab) (C, D) in the hippocampal CA1 region of AD patients. Nuclear morphology indicates that most co-localized QUIN and TDO immunoreactive cells are neurons, with some granular QUIN^+^ labelling distributing around cytoplasmic TDO^+^ labelling (A). Tau positive labelled tangles co-localized with TDO-positive neurons (B). Some diffuse 4G8-immunoreactive deposits appeared around blood vessels (C). TDO-positive cells appeared within almost all the Aβ deposits, with distribution in the centre and periphery of the deposits (D).

Co-localized TDO- and QUIN-positive cells were found mainly in hippocampal and subicular pyramidal neurons of AD patients. TDO staining in neurons was cytoplasmic, whereas QUIN staining was located in vacuoles either on the neuronal membrane or within the cytoplasm (Fig. 9A). Tau-positive neurons were found mainly in CA1 and subiculum of AD patients. Tau positive staining in the form of fully formed tangles co-localized with TDO immunostaining (Fig. 9B). Diffuse 4G8-immunoreactive deposits were widely detected in all AD patients, spreading out from the CA1 to CA3 and subiculum regions, with some deposits appearing around blood vessels (Fig. 9C). TDO-positive cells appeared within almost all 4G8-positive deposits (Fig. 9C, D).

## Discussion

The KP is the primary pathway for the metabolism of the essential amino acid Trp in mammalian tissues, including the brain [19]. Activation of IDO-1 and/or TDO, the initial enzymes of the KP, leads to the formation of several downstream catabolites with neurotoxic and/or neuroprotective effects [20]. IDO-1 plays a key role in immune tolerance [21] and its activation has been implicated in the pathogenesis of neuroinflammatory and neurodegenerative disorders [20]. IDO-1 has attracted attention due to its potential as a target in the treatment and prevention of human diseases. Because of its predominant hepatic expression, TDO has always been thought to mainly contribute to systemic Trp metabolism. Recent and increasing evidence suggest that TDO may also play important roles in both mouse [22]–[24] and human brain functions [8], [25]. However, the physiological and pathophysiological roles of TDO in the CNS have not been explored.

To elucidate the involvement of TDO in the pathogenesis of AD, this study aimed to determine mRNA expression of TDO in 4 brain regions in 3 age groups of 3xTg AD mice compared to age-matched WT controls. We demonstrated that in the cerebellum, the levels of expression of TDO mRNA were significantly increased in 3xTg AD mice at 6–8 months and 10–12 months. In contrast, IDO-1mRNA was not found to be significantly higher in the same brain regions of 3xTg AD mice compared to WT controls. We also confirmed the expression of TDO at protein level in AD brain by immunohistochemistry and western blot analysis. We found that TDO immunoreactivity increased with age in both 3xTg AD and WT mice. However, there was a significantly higher density of TDO-positive cells in 3xTg AD adult mice. Western blot analysis also demonstrated significantly higher levels of TDO in the cerebellum of 8-month-old 3xTg AD, compared to WT mice. More interestingly, this increase of TDO in AD mouse brain was also found in human AD brain. A significantly higher TDO and IDO-1 immunoreactivity was observed in the hippocampus of 4 patients with AD when compared to 4 age and sex-matched normal controls. Further investigation is necessary to clarify the TDO and IDO1 expression in the cerebellum region of human AD brains.

Our data show an increased consumption of Trp in the hippocampal and cerebellar regions of AD brain reflecting higher TDO/IDO-1 activity. We did not find significant increase in levels of KYN or KYNA in the same regions suggesting that KYN is preferentially and rapidly metabolized along the QUIN/PIC pathways. This is supported by the observation of up-regulated expression of 3HAAO, ACMSD and QPRT mRNA in the cerebellum of 3xTg AD brain. The enhanced expression of ACMSD in the cerebellum and hippocampus led to the high concentration of PIC [18] in both regions in AD mouse brain, in particular, in young adult and old groups. This may be explained by higher metabolism in young adult mice and neuroinflammation in older AD mice. Highly up-regulated 3HAAO also increases QUIN synthesis. One of the key results is that the highest levels of QUIN were only found in the hippocampus, the most vulnerable area in AD, but not in the cerebellum of AD mouse brain. This may be accounted for by the strong expression of QPRT in the cerebellum leading to a significant catabolism of QUIN toward the biosynthesis of NAD^+^ in this region.

In addition to TDO/IDO-1, there was also a pronounced age-related increase in the expression of the downstream KP enzymes in WT mice brain (data not shown). Increased IDO-1 mRNA has been previously reported in aged BALB/c mice and is suggested to be associated with age-associated priming of microglia [26]. In mouse brain, Trp and KYN levels decrease with ageing (Miura et al., 2008). Trp degradation is increased in the peripheral blood of AD patients and age-matched controls, compared to the younger controls: however these changes were more prominent in the AD groups compared to age-matched control group [12]. Our results add more evidence to the potential influence of ageing exacerbating Trp metabolism.

PIC is the main endogenous metal chelator within the brain [27]. PIC is an endogenous neuroprotective compound mostly produced by neurons [18], [28]. In nanomolar concentrations, PIC protects against QUIN- and kainic acid-induced neurotoxicity [29]. Unlike KYNA and other NMDA receptor antagonists, PIC can effectively block QUIN neurotoxicity but without affecting the excitatory effect. The mechanism by which PIC attenuates QUIN neurotoxicity is still unknown. We detected high levels of PIC in both hippocampus and cerebellum of old 3xTg AD mice but unlikely to be sufficient to block QUIN excitotoxicity. On the other hand, PIC can exacerbate the immune response by increasing chemokine release [30] and can even be neuro- and gliotoxic [31]. PIC displays Janus face type effects and its functions in AD remain to be determined.

Within the CNS, IDO-1 has been shown to express in murine microglia, neurons, astrocyte and vascular endothelium upon treatment with IFN-γ [32], [33]. We have previously shown that *in vitro* IDO-1 is expressed in human primary neurons, astrocytes and microglia upon IFN-γ stimulation [34] but that it is missing in the oligodendrocyte [35]. In human AD hippocampus, IDO-1 is expressed in astrocytes and microglia and to a lesser extent in neurons [14]. A recent study has described TDO being expressed in mouse brain mostly in neurons, some astrocytes, but not in microglia [24]. In agreement with this work, we found that in 3xTg AD brain most of the TDO immuno-positive cells were neurons and some astrocytes. We also detected TDO in murine microglia and in the Purkinje cell layer (data not shown) but not in mature granule cells of the adult mouse dentate gyrus in the hippocampus or granule cells in the cerebellum region. Consistent with Miller et al [8], we have shown that in the hippocampus of human AD brain, TDO is mostly expressed by neurons, occasionally by astrocytes, but not by microglia (data not shown).

In terms of KP metabolites in human brain cells, QUIN is only produced by microglia [36], [37] whereas astrocytes produce large amounts of KYN and to a lesser extent KYNA [38]. Human primary neurons mostly produce PIC [18]. ACMSD is considered as a switch in the KP favouring production of PIC instead of QUIN [39]. We have previously demonstrated that QUIN is taken up and accumulates in human cortical neurons [14] [15] and that IDO-1 and TDO are co-expressed in these cells [18]. The present study confirms the co-localization of TDO and QUIN in neurons in the hippocampus of AD patients and we showed that QUIN staining is located in vesicles together with TDO cytoplasmic expression. The exact role of TDO in neurons in AD brain remains unclear but is likely to be associated with PIC production, possibly aiming to counteract QUIN effects. This hypothesis is supported by our results showing that TDO expression is associated with both NFTs and senile plaques. We previously showed that QUIN production is increased within senile plaque [40] and is able to increase tau hyperphosphorylation [15]. Aβ1-42 can induce IDO-1 expression leading to QUIN over-production by human primary microglia [41]. The combination of chronic exposure to Aβ1-42 and the increased level of IFN-γ, associated with the neuroinflammatory state, could be involved in the progression of AD [42].

The increase in TDO expression in AD remains to be explored. TDO is not activated by inflammatory cytokines [43] but only by its substrate (trp) and hormones such as cortisol and prolactin [44]. Trp catabolism is increased in the blood of AD [12], which is in accordance with our data showing low levels of Trp in most of the brain regions in 3xTg AD mice. So Trp is unlikely to induce TDO in AD. However, the activation of the KP can lead to a decrease of serotonin levels that is associated with a diminution of the inhibitory effect on the amygdaloidal complex and an increased production of cortisol [45]. Prolactin is also increased in AD brain [46]. Furthermore, some inflammatory mediators such as prostaglandins may also be involved in the modulation of the action of hormones that are known to be elevated in affected regions of AD brain [47]. Therefore it is tempting to speculate that the increased hormonal response could lead to the augmentation of TDO expression in AD brain.

Currently no medication has been able to significantly slow down the progression of AD. Inhibitors of KP enzymes have already been investigated as a therapeutic approach for AD. A recent study has confirmed a direct link between Trp metabolism in the blood and neurodegeneration by using an inhibitor of kynurenine 3-monooxygenase in a monotransgenic AD mouse model [48]. Future work using TDO [49] and/or IDO-1 inhibitors [50] in AD mice could also provide a new therapeutic doors for AD.

In conclusion, we have demonstrated for the first time that TDO is over-expressed in the brain of 3xTg AD mice and human AD patients. We have shown that TDO cellular expression is mainly in neurons and in some astrocytes, in both human and mouse brains. This study also provides further evidence that elevated TDO and IDO-1 expression leads to the excessive formation of KP metabolites such as QUIN and/or PIC, which are likely to be involved in the neurodegenerative processes in AD.
